# Transplantation of novel human GDF5-expressing CHO cells is neuroprotective in models of Parkinson's disease

**DOI:** 10.1111/j.1582-4934.2012.01562.x

**Published:** 2012-09-26

**Authors:** Daniel J Costello, Gerard W O'Keeffe, Fiona M Hurley, Aideen M Sullivan

**Affiliations:** a Department of Anatomy and Neuroscience Biosciences Institute, University College CorkCork, Ireland; b Department of Neurology, Cork University HospitalWilton, Cork, Ireland

**Keywords:** growth/differentiation factor 5, neurotrophic factor, Parkinson's disease, neurodegeneration, dopamine

## Abstract

Growth/differentiation factor 5 (GDF5) is a neurotrophic factor that promotes the survival of midbrain dopaminergic neurons *in vitro* and *in vivo* and as such is potentially useful in the treatment of Parkinson's disease (PD). This study shows that a continuous supply of GDF5, produced by transplanted GDF5-overexpressing CHO cells *in vivo,* has neuroprotective and neurorestorative effects on midbrain dopaminergic neurons following 6-hydroxydopamine (6-OHDA)-induced lesions of the adult rat nigrostriatal pathway. It also increases the survival and improves the function of transplanted embryonic dopaminergic neurons in the 6-OHDA-lesioned rat model of PD. This study provides the first proof-of-principle that sustained delivery of GDF5 *in vivo* may be useful in the treatment of PD.

## Introduction

Parkinson's disease (PD) is a common neurodegenerative disease characterized by motor symptoms that are caused by the progressive degeneration of dopaminergic neurons projecting from the substantia nigra (SN) to the striatum. Currently, the mainstay therapy of PD involves exogenous L-dopa or dopamine receptor agonists, but these treatments become less effective as the disease progresses (for review, see [1]). Alternative experimental treatment strategies include the use of neuroprotective agents to rescue the remaining dopaminergic neurons, and the replacement of the lost dopaminergic neurons by transplantation of embryonic midbrain [ventral mesencephalon (VM)] tissue ectopically in the striatum. While many studies have shown that VM transplants can survive, integrate and improve motor function in patients (for reviews see [1–6]), significant obstacles need to be overcome, before this approach is widely used for PD therapy (for recent reviews, see [7, 8]). In addition to the need for standardization and optimization of surgical methodology and patient selection, there is a critical problem regarding the survival of the transplanted cells. Only 5–10% of the dopaminergic neurons survive the grafting procedure [4, 9], and several agents are under investigation for their potential to improve dopaminergic neuronal survival after transplantation (for reviews, see [10–13]). Neurotrophic factors are proteins that play important roles to support specific neuronal populations in the developing brain, and also have protective actions on some populations of mature neurons. Neurotrophic factors that have such effects on dopaminergic neurons are being studied by many groups with regards to their potential to improve the survival of transplanted dopaminergic neurons, as well as to prevent the ongoing degeneration of nigrostriatal neurons in the parkinsonian brain (for reviews see [11, 14, 15]).

Growth/differentiation factor 5 (GDF5) is a member of the transforming growth factor (TGF)-β superfamily of proteins [16]. In the embryonic rat midbrain, GDF5 expression correlates temporally with the differentiation of dopaminergic neurons, peaking at embryonic day (E) 13-14 [17], around the time when these cells are undergoing the final stages of their division and maturation [18, 19]. GDF5 treatment increases the survival of dopaminergic neurons in cultures of embryonic rat VM, and improves their morphological development [20–22]. Furthermore, GDF5 treatment attenuates the dopaminergic neurotoxicity induced by either the neurotoxin *N*-methyl pyridinium ion (MPP^+^) [23] or free radical donors [24] in cultures of E14 rat VM. *In vivo*, intracerebral administration of GDF5 confers neuroprotective and neurorestorative effects on the adult rat nigrostriatal pathway in 6-hydroxydopamine (6-OHDA)-lesioned adult rat models of PD [25–27]. Furthermore, GDF5 treatment increases the survival of embryonic rat dopaminergic neurons after intrastriatal transplantation in 6-OHDA-lesioned adult rats [21, 28]. However, the effects of long-term exposure to GDF5 on E14 VM transplants or on the host adult rat brain have not been examined. As GDF5 has been proposed as a candidate neurotrophic factor for PD treatment, information on its sustained delivery is necessary. This study set out to examine the effects of GDF5 production by a GDF5-overexpressing CHO cell line, after intrastriatal implantation and after co-implantation with E14 rat VM grafts in 6-OHDA-lesioned adult rat models of PD. This GDF5 production *in vivo* was found to have neuroprotective and neurorestorative effects on the host nigrostriatal system and to improve the survival and morphology of the co-transplanted VM dopaminergic neurons. These data corroborate previous studies showing neuroprotective and restorative effects of GDF5 in adult rat models for PD, and support the future development of appropriate delivery systems to achieve long-term and targeted delivery of this neurotrophic factor to the damaged nigrostriatal pathway in this disease.

## Materials and methods

### Cell culture and western blotting

A CHO cell line (‘GDF5-CHO’) stably transfected with the human GDF5 gene, and a CHO cell line (‘mock-CHO’) stably transfected with an empty plasmid were kind gifts from Biopharm GmbH, Heidelberg, Germany. The GDF5-CHO cell line was invented and subsequently patented [European patent: EP 0 866 125 A1] by Hoechst Marion Roussel, Ltd., Tokyo 107-8465, Japan. This involved the ligation of a DNA fragment containing the human GDF5 gene into a pAB stop vector; the resulting vector was designated pMS99. Ten micrograms of pMS99 and 2 μg of pSV0Adhfr were dissolved in 1 ml of 25 mM HEPES, 140 mM NaCl, 0.75 mM Na_2_HPO_4_, then mixed with 50 μl of 2.5 M CaCl_2_. This mixture was applied to CHO cells for 30 min. The cells were cultured in MEMα^+^ (MEM-alpha containing ribo- and deoxyribonucleosides) containing 10% foetal bovine serum (FBS) for 4–6 h at 37°C. The cells were treated with 10% glycerol in MEMα^+^ containing 10% FBS for 3 min and then cultured for 2 days in MEMα^+^ containing 10% FBS. The cells were placed in MEMα without ribo- and deoxyribonucleosides (MEMα^−^) containing 10% dialysed FBS for selection of the transformants. Clones which produced high levels of GDF5 were selected stepwise in increasing concentrations of methotrexate to amplify the GDF5 gene in accordance with the pSV0Adhfr. The CHO cell line with the highest GDF5 production (MC-2) was selected and further cultured in MEM-α with 10% foetal calf serum (FCS), 2 mM l-glutamine, 400 nM methotrexate, 100 U/ml penicillin, 100 μg/ml streptomycin (all from Sigma-Aldrich, Arklow, Wicklow, Ireland) until confluent. For GDF5 production, the cells were cultured in DMEM/Ham's F12, supplemented with 10 KIU aprotinin, 1 mM sodium butyrate, 6.7 ng/ml sodium selenate, 5.5 μg/ml transferrin, 2 μg/ml ethanolamine, 9 μg/ml insulin, 100 U/ml penicillin, 100 μg/ml streptomycin (all from Sigma-Aldrich). Every day, two-thirds of this serum-free medium were harvested and stored at −20°C. Western blotting was performed on conditioned medium samples and CHO cell lysates, as previously described [17]. Cell lysates were prepared by incubation of cells in lysis buffer (50 mM Tris pH 7.4, 150 mM NaCl, 10% glycerol, 1% Triton X-100, 1 mM EDTA, 0.32 M sucrose, 100 μg/ml PMSF, 1 μg/ml bacitracin, 1 μg/ml pepstatin A, 1 μg/ml aprotinin, 1 μg/ml leupeptin and 1 μg/ml antipain) on ice for 1 h. Insoluble debris was removed by centrifugation at 10,000 × *g* for 10 min at 4°C. Protein concentrations were determined using a Bradford assay with bovine serum albumin (BSA), as a standard. Sample extracts containing 30 μg of protein or 15 μl of conditioned medium were diluted 1:1 in a reducing sample buffer [7 M urea, 0.1% sodiumphosphate, 1% sodium dodecyl sulphate (SDS), 0.01% bromophenol blue, 100 mM dithiothreitol], and samples were separated on a 12% SDS-polyacrylamide gel and blotted to PVDF membranes. Recombinant human GDF5 (50 ng) was run as a positive control. Blots were incubated in either of the primary antibodies, MP-52 (which recognizes monomeric GDF5) or aMP-5 (which recognizes dimeric GDF5; both kindly provided by Biopharm GmbH), for 12–16 h in 1% BSA dissolved in 10 mM PBS containing 0.1% Tween20 (PBS-T). Following three washes for 10 min in PBS-T, blots were incubated in horseradish peroxidase (HRP)-labelled anti-rabbit or anti-mouse IgG (1:2000; Promega, Southampton, UK). Membranes were rinsed three times for 10 min with PBS-T and developed with ECL Plus (GE Healthcare Bio-Sciences, Buckinghamshire, UK).

Cultures of E14 rat VM were prepared as previously described [20]. A total volume of 4 ml of conditioned medium harvested from GDF5-CHO cells at 2 days *in vitro* (DIV) was concentrated to 500 μl, with molecular weight cut-off filters (Millipore, Watford, UK). The concentrated medium samples were added directly to the medium of E14 rat VM cultures at a final dilution of 1:10. Control cultures received medium from mock-CHO cells. The cultures of E14 rat VM were grown for a further 3 DIV before being fixed and processed for immunocytochemistry for tyrosine hydroxylase (TH; 1:300; rabbit polyclonal; Millipore) or β-III tubulin (1:300, mouse monoclonal; Promega), visualized with HRP-linked secondary antibodies and counter-stained with DAPI (1:5000; Sigma-Aldrich).

### Lesion surgery and behavioural testing

Female Sprague–Dawley rats, approximately 3 months old and weighing 200–270 g, were used for all experiments (Biological Services Unit, UCC). In each experiment, there were five rats in each treatment group. Each rat received either a complete unilateral lesion of the left medial forebrain bundle (MFB) or left striatum, as previously described [25, 26]. Briefly, rats were anaesthetized with a mixture of ketamine (80 mg/kg, i.p.) and xylazine (5 mg/kg, i.p.). Surgery was carried out with a Kopf® stereotaxic frame (Tajunga, CA, USA) with the incisor bar set at 5.0 mm above the interaural line and the co-ordinates taken from bregma and dura [29]. For MFB lesions, 6-OHDA (8 μg as free base in 4 μl 0.9% saline with 0.1% ascorbic acid) was injected at the following co-ordinates: AP: −2.2, LV: +1.5, DV: −7.9. For striatal lesions, 6-OHDA (20 μg as free base in 3 μl 0.9% saline with 0.1% ascorbic acid) was injected at the following co-ordinates: AP: +1.0, LV: +3.0, DV: −5.0. Following surgery, animals were housed singly in an environmentally controlled room with free access to food and water. At various time-points, rotational behaviour was assessed after injection of (+)-amphetamine sulphate (5 mg/kg, i.p., Sigma-Aldrich). Rotations were recorded over 60 min beginning 5 min after amphetamine injection and are expressed as the mean of full body ipsiversive turns minus contraversive turns.

### Transplantation and immunosuppression

For the transplant experiments, either mock-CHO or GDF5-CHO cells were stereotaxically injected into either the striatum at the same time as 6-OHDA injection into the ipsilateral MFB or into the striatum or SN at 7 days after intrastriatal 6-OHDA injection (5 × 10^5^ cells divided equally between three sites). The following co-ordinates were used (incisor bar at +5.0 mm): SN: AP −3.0, ML +2.5, DV −8.5, −7.5, −6.5; striatum: AP +1.0, LV +3.0, DV −5.0, −4.0, −3.0. For co-transplantation experiments, a suspension of 5 × 10^5^ E14 VM cells was prepared as previously described [28], mixed with 5 × 10^5^ mock-CHO or GDF5-CHO cells and transplanted stereotaxically into the left striatum at 7 days after 6-OHDA injection into the ipsilateral MFB, divided equally between three sites at the co-ordinates described above. For GDF5-treated grafts, 50 μg rhGDF5 (a gift from Biopharm GmbH) was mixed with the E14 cell suspension prior to transplantation. For the ‘sham graft’ group, an equivalent volume of saline was injected using identical surgical procedures and co-ordinates as used for the grafting groups. For immunosuppression, rats (where indicated) received oral cyclosporin A (Cy-A-Neoral®) at a dose of 5 mg/kg/day for 5 days prior to transplantation and daily until killed.

### Immunohistochemistry

All animals were transcardially perfused with 4% paraformaldehyde (Sigma-Aldrich) in PBS, the brains were post-fixed by immersion, 4% paraformaldehyde for 24 h, then cryopreserved in 30% sucrose. Coronal 10 μm sections were collected through the entire region of the striatum with a cryostat (Leica M1900, Laboratory Instruments and Supplies, Ashbourne, Ireland) and processed for immunohistochemistry as previously described [25], using the following primary antibodies: GDF5 (aMP-5; 1:200, Biopharm), TH (1:1000, Millipore), MHC I (1:400; Serotec, Serotec Ltd, Kidlington, Oxford, UK), MHC II (1:400; Serotec), CD 4 (1:400; Serotec), CD 8 (1:400; Serotec), ED 1 (1:400; Serotec), IgM (1:200; Sigma-Aldrich) and visualized using HRP-linked secondary antibodies.

### Data analysis

For analysis of the cellular phenotypic composition of primary cultures following treatment with conditioned medium, four random fields were analysed per culture well. Total cell number and number of immunoreactive cells per field were quantified. Data are presented as mean ± S.E.M. of 12 wells from three independent experiments. Statistical evaluation was performed using Student's *t*-test, with significance set at *P* < 0.05.

Quantification of dopaminergic neurons in the host midbrain and in the grafts was performed with the unbiased Physical Paired Dissector and Cavalieri stereological methods [30], as described previously [21]. In brief, pairs of sections from each of three levels through the midbrain (AP −4.8, −5.6 and −6.4 relative to bregma; [31]) were examined under 20× objective with an Olympus-X70 fluorescence microscope. Representative fields (three per SN, per hemisphere) were photographed and cells were counted by a blinded observer. The unbiased Physical Paired Dissector method was used to measure the numerical density of TH-immunopositive neurons in each SN [30]. For the MFB and striatal lesion studies, data are presented as the number of TH-immunopositive neurons in each lesioned SN as a percentage of that in the intact SN of the same animal. For the graft study, data are presented as the number of TH-immunopositive neurons in the grafted striatum post-mortem as a percentage of the estimated number of dopaminergic neurons in the original graft (assumed to be 10% of the total number of grafted cells).

Quantification of the immune response in the host striatum was performed with a rating scale, as previously described [32]. Statistical evaluation of normally distributed data (parametric data) was carried out with univariate (anova), and multivariate analysis of variance (manova) with *post-hoc* Tukey's testing. Significance was set at *P* < 0.05.

## Results

### Conditioned medium from a GDF5-overexpressing CHO cell line increases the number of dopaminergic neurons in cultures of E14 rat VM

**Fig 1:**
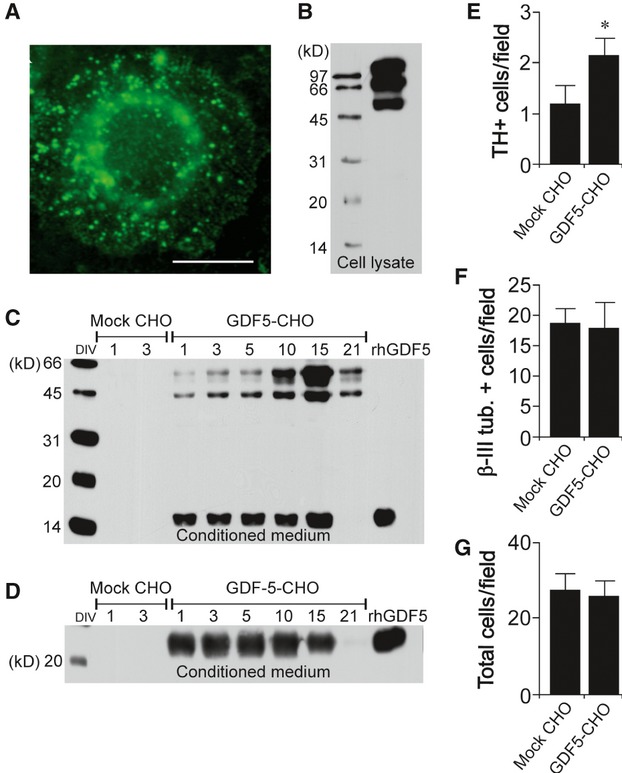
Characterization of GDF5 expression and secretion by the GDF5-CHO cell line *in vitro*. (A) Representative photomicrograph of a single GDF5-CHO cell grown for 3 DIV, immunocytochemically stained for GDF5. Scale bar = 10 μm. (B) Western blot detection (using MP52 antibody) of high molecular weight GDF5 precursor proteins in a GDF5-CHO cell extract after 15 DIV. (C) Western blot detection (run under reducing conditions, using MP52 antibody) of GDF5 monomeric proteins in conditioned medium from mock-CHO and GDF5-CHO cell cultures after 1, 3, 5, 10, 15, and 21 DIV, as indicated. (D) Western blot detection (run under non-reducing conditions, using aMP5 antibody) of GDF5 dimeric proteins in conditioned medium from mock-CHO and GDF5-CHO cell cultures after 1, 3, 5, 10, 15, and 21 DIV, as indicated. rhGDF5 was run as a positive control in both (C) and (D). Graphical representation of the number of (E) dopaminergic (TH-positive) neurons, (F) total (β-III tubulin-positive) neurons and (G) total cells per field in E14 rat VM cultures treated with conditioned medium from mock-CHO or GDF5-CHO cells for 3 DIV. Data are shown as mean ± SEM (*P < 0.05 compared with mock-CHO cell group; Student's *t*-test; *n* = 3 experiments).

To achieve long-term expression of GDF5 *in vitro* and *in vivo,* a CHO cell line that had been stably transfected with the human GDF5 gene (GDF5-CHO) was used. Firstly, this cell line was characterized *in vitro*. GDF5-CHO cells were cultured for 3 DIV before being fixed and processed for immunocytochemistry. Confocal microscopy showed that these cells displayed abundant intracellular immunoreactivity for GDF5, which was predominantly localized in vesicles (Fig. 1A). To confirm that these cells produced mature GDF5 protein, Western blotting was performed on cell lysates and on conditioned medium from these cultures. In cell lysates, a number of high molecular weight bands were detected, one of which had a molecular weight of 55 kD (Fig. 1B), which corresponds to that of the monomeric form of the GDF5 precursor protein. The other high molecular weight bands correspond to various processed (dimeric) forms of the precursor protein. Mature GDF5 was not detected intracellularly (Fig. 1B). To determine whether these cells secreted mature GDF5, conditioned medium samples from mock-CHO and GDF5-CHO cells were examined under reducing conditions. Mock-transfected cells did not secrete GDF5 (Fig. 1C), whereas GDF5-CHO cells secreted both GDF5 precursor proteins and mature (monomeric; 15 kD) GDF5 into the medium for at least 15 DIV (Fig. 1C). These cells continued to secrete GDF5 precursor proteins after 21 DIV, but did not secrete mature GDF5 protein at this time-point (Fig. 1C). To confirm the presence of biologically active (dimeric; 25 kD) GDF5, Western blotting was performed under non-reducing conditions using an antibody (aMP5) that recognizes the dimeric form of GDF5. This showed that the GDF5-CHO cells secreted mature GDF5 for up to 15 DIV, whereas mock-CHO cells did not produce dimeric GDF5 at any point (Fig. 1D).

To examine the effects of GDF5 secreted from the CHO cell line on dopaminergic neurons *in vitro*, E14 rat VM cultures were treated with conditioned medium from either GDF5-CHO or mock-CHO cells for 3 DIV. A pilot study on E14 rat VM cultures had demonstrated that mock-CHO cell conditioned medium had no effect on the number of TH-immunopositive cells, β-III tubulin-positive cells or total number of cells, compared with control medium. Immunocytochemical analysis showed that treatment with the GDF5-CHO conditioned medium resulted in a twofold increase in the numbers of dopaminergic neurons per field after 3 DIV, compared with treatment with the mock-CHO conditioned medium (Fig. 1E; *P* < 0.05). Treatment with GDF5-CHO conditioned medium did not have any significant effect on the total numbers of neurons per field (Fig. 1F) or the total number of cells per field (Fig. 1G), compared with those in cultures treated with mock-CHO conditioned medium.

### Characterization of the host immune responses to CHO cell xenografts and to E14 rat VM allografts in the adult rat striatum

**Fig 2:**
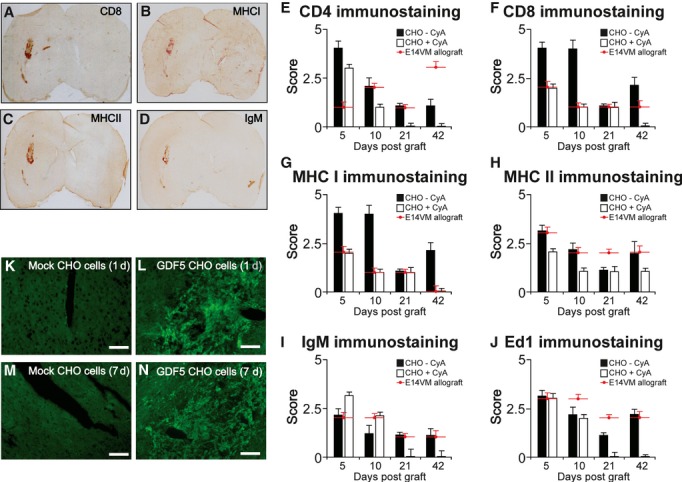
Characterization of GDF5 expression by the GDF5-CHO cell line *in vivo* and of the immune response of the host striatum to GDF5-CHO cell transplantation. (A)–(D) Representative photomicrographs showing (A) CD 8, (B) MHC I, (C) MHC II and (D) IgM immunoreactivity at the graft sites at 10 days after intrastriatal grafting of CHO cells. Graphical representation of the extent of the immunoreactivity to (E) CD 4, (F) CD 8, (G) MHC I, (H) MHC II, (I) IgM and (J) Ed 1 in the adult rat striatum at 5, 10, 21 and 42 days after grafting of E14 VM cells, or CHO cells with or without oral CyA, as indicated. (K)–(N) Representative photomicrographs showing intrastriatal transplants of mock-CHO cells and GDF5-CHO cells at 1 and 7 days post-grafting, as indicated, immunocytochemically stained for GDF5 using aMP5 antibody. Scale bar = 100 μm.

Prior to characterizing the functional effects of intrastriatal implantation of GDF5-CHO cell xenografts in PD models, the host immune response to these xenografts was examined in unlesioned adult rats and compared with that response to allografts of E14 rat VM. Furthermore, the effect of oral administration of CyA on the host response to CHO cell grafts was characterized. Suspensions of 5 × 10^5^ E14 rat VM cells or 5 × 10^5^ CHO cells were implanted into the striata of adult rats. A third group of rats received intrastriatal grafts of CHO cells as well as treatment with oral CyA (5 mg/kg/day) for the previous 5 days and every day thereafter until being killed. At 5, 10, 21 and 42 days post-lesion, groups of animals were perfused and processed for immunocytochemistry. There was a robust host immune response, as evidenced by strong immunoreactivity for CD 4, CD 8, MHC I, MHC II, IgM and ED 1 around the graft site in the striatum of each rat that had received a CHO xenograft without CyA (Fig. 1A–D). A weaker immmue response was observed in the striata of rats that had received CyA treatment as well as a CHO xenograft, and in those that had received an E14 VM allograft. This immunoreactivity was quantified using a rating scale from 0 (no immunostaining) to 4 (very dense immunostaining in and around the graft) [32]. In general, the xenografts elicited a more robust host immune response than the allografts, but oral administration of CyA significantly reduced the extent of this response to equal or lower levels as that elicited by the allografts (Fig. 2E–J).

### GDF5-overexpressing CHO cells secrete GDF *in vivo* after implantation into the adult rat striatum

To establish whether grafts of GDF5-CHO cells could produce GDF5 protein *in vivo*, 5 × 10^5^ mock-CHO or GDF5-CHO cells were implanted into the striata of adult rats and the brains of these animals were processed for GDF5 immunohistochemistry at 1, 3, 5 and 7 days post-transplantation. Strong expression of GDF5 was seen in the striatum of each rat that had received a GDF5-CHO graft, at all time-points examined (Fig. 2L and N). No GDF5 immunoreactivity was detected in striata of rats that had received mock-CHO cells (Fig. 2K and M). The immunostaining was maximal in the region of the implant, with very little staining in other areas.

### Transplantation of GDF5-overexpressing CHO cells confers neuroprotective and neurorestorative effects on adult rat midbrain dopaminergic neurons

**Fig 3:**
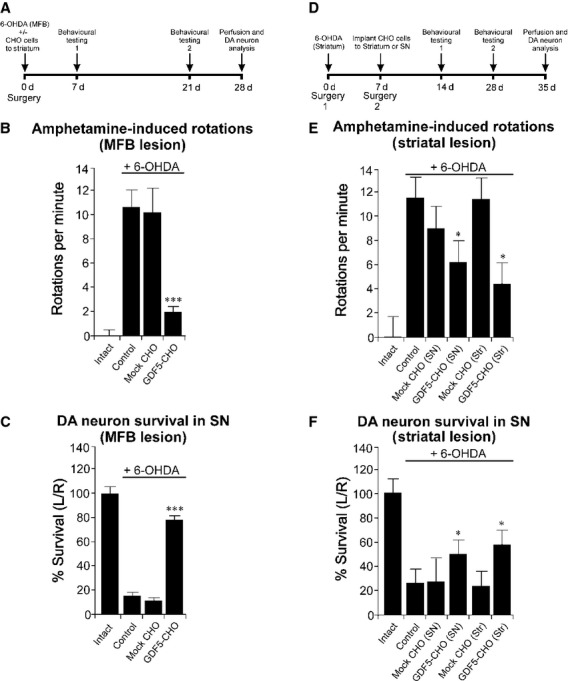
Neuroprotective and neurorestorative effects of GDF5-CHO cells in two adult rat models of PD. (A) Outline of experiment to analyse the neuroprotective effect of GDF5-CHO cells in the 6-OHDA MFB lesion model of PD. (B) Amphetamine-induced rotational rates at 21 days post-surgery for each treatment group (*n* = 5 per group). (C) Survival of dopaminergic neurons in the SN at 28 days post-surgery expressed as the number of TH-positive neurons in the left SN as a percentage of those in the right. Data are expressed as mean ± SEM. ***P < 0.001 compared with control (lesion only) and mock-CHO groups, anova with *post-hoc* Tukey's test. (D) Outline of experiment to analyse the neurorestorative effect of GDF5-CHO cells in the 6-OHDA striatal lesion model of PD. (E) Amphetamine-induced rotational rates at 28 days post-surgery for each treatment group (*n* = 5 per group). (F) Survival of dopaminergic neurons in the SN at 35 days post-surgery, expressed as the number of TH-positive neurons in the left SN as a percentage of those in the right. Data are expressed as mean ± SEM. *P < 0.05 compared with control (lesion only), mock-CHO (SN) and mock-CHO (striatum) groups; anova with *post-hoc* Tukey's test.

To investigate whether the GDF5-CHO cells could ameliorate the effects of a 6-OHDA lesion of the adult rat MFB, groups of rats received oral CyA for 5 days prior to receiving an injection of either 5 × 10^5^ mock-CHO cells or 5 × 10^5^ GDF5-CHO cells into the left striatum, in addition to a 6-OHDA injection into the left MFB (Fig. 3A). At 7 and 21 days after lesion surgery, animals that had received only a 6-OHDA lesion displayed rotation rates of more than eight ipsiversive turns per minute, which indicates almost complete ablation of the ipsilateral nigrostriatal pathway [33]. Those animals that had received a transplant of mock-CHO cells had rotational rates that were not different from animals that had received a 6-OHDA lesion only, whereas those that had received a graft of GDF5-CHO cells displayed significantly lower amphetamine-induced rotational rates than the lesion-only and mock-CHO cell groups (*P* < 0.001; Fig. 3B). Immunohistochemical analysis showed that those animals that had received a graft of GDF5-CHO cells had significantly higher numbers of dopaminergic neurons in the left SN (expressed as a percentage of those in the right SN) at 4 weeks post-lesion, when compared with lesion-only and mock-CHO cell groups (*P* < 0.001; Fig. 3C).

A further study used the intrastriatal 6-OHDA lesion model to determine whether GDF5-CHO cells transplanted to the striatum or SN at 1 week after the lesion could affect the ongoing degeneration of the nigrostriatal pathway (Fig. 3D). Animals received oral CyA for 5 days prior to receiving an injection of 6-OHDA into the left striatum. At 1 week after surgery, these animals received a graft of 5 × 10^5^ mock-CHO cells or 5 × 10^5^ GDF5-CHO cells into the left striatum or left SN. At 28 days after lesion surgery, amphetamine-induced rotational rates of at least eight turns per minute were displayed by all rats that had received a lesion only, and those that had received mock-CHO cells into either the SN or striatum (Fig. 3E). Those animals that had received a graft of GDF5-CHO cells into either the striatum or SN displayed significantly lower rotational rates compared with the lesion-only and mock-CHO cell (either striatum or SN) groups (*P* < 0.05; Fig. 3E). Immunohistochemistry showed that the animals that had received a graft of GDF5-CHO cells into either the striatum or SN had significantly higher numbers of dopaminergic neurons in the left SN at 4 weeks post-transplantation, when compared with lesion-only and mock-CHO (either striatum or SN) cell groups (*P* < 0.05; Fig. 3F).

### Co-transplantation of GDF5-overexpressing CHO cells increases the survival of E14 rat VM grafts in an adult rat model of PD

**Fig 4:**
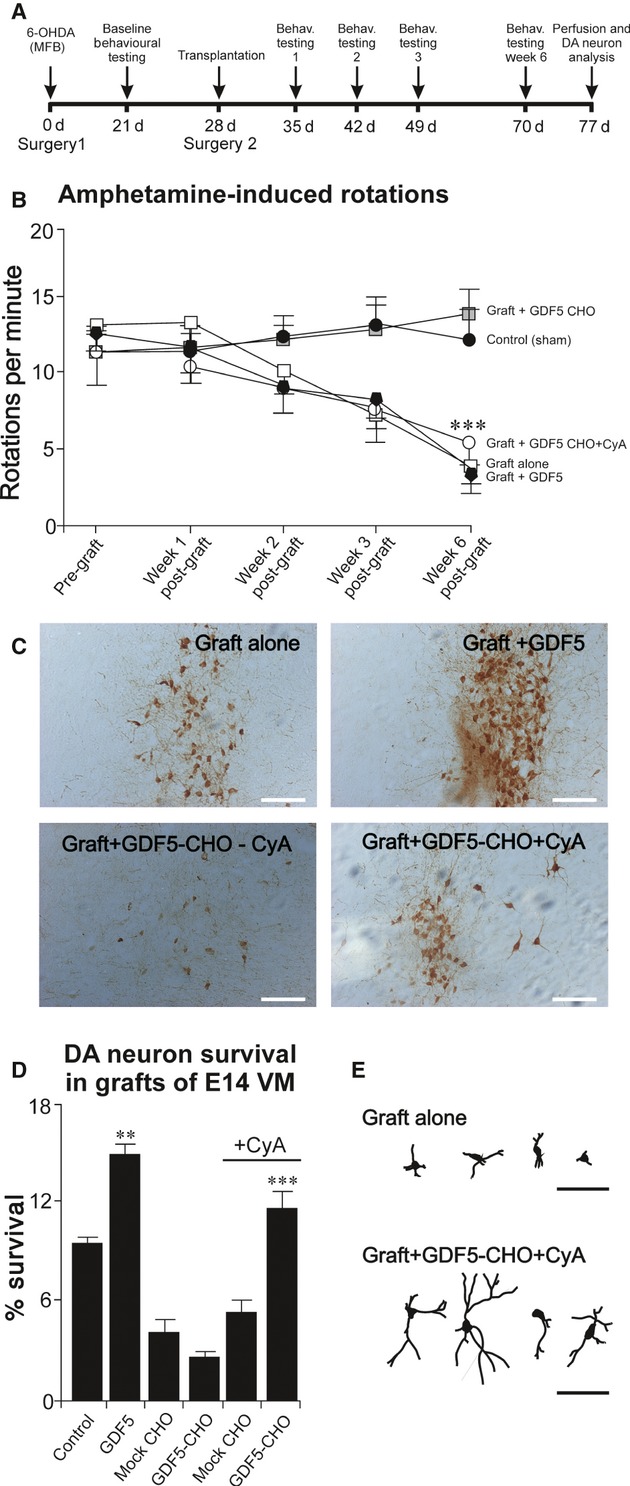
GDF5-CHO cells improve the survival and function of grafts of E14 rat VM in an adult rat model of PD. (A) Outline of experiment to analyse the effects of co-grafting GDF5-CHO cells with E14 rat VM grafts in the 6-OHDA MFB lesion model of PD. (B) Amphetamine-induced rotational rates at 3 weeks after lesion surgery and at 1, 2, 3 and 6 weeks post-grafting surgery for each treatment group (*n* = 5 per group). (C) Representative photomicrographs of grafted TH-positive neurons in the ‘graft alone’, ‘graft + GDF5’, ‘graft + GDF5-CHO without CyA’ and ‘graft + GDF5-CHO with CyA’ groups at 7 weeks post-grafting. (D) Survival of grafted dopaminergic neurons (expressed as a percentage of the total number grafted) at 7 weeks post-grafting in each treatment group (*n* = 5 per group). Data are expressed as mean ± SEM. (**P < 0.01, ***P < 0.001 *versus* control (‘graft alone’), GDF5-CHO without CyA, mock-CHO with or without CyA groups; anova with *post-hoc* Tukey's test). (E) Representative line drawings showing individual TH-positive neurons in control (‘graft alone’) and treated ‘graft + GDF5-CHO with CyA’ grafts. Scale bar = 100 μm.

The next experiment investigated whether the sustained delivery of GDF5 *in vivo* could improve the survival and function of intrastriatal grafts of E14 rat VM in adult rats that had received a 6-OHDA lesion of the MFB (Fig. 4A). At 3 weeks after the lesion, all rats displayed amphetamine-induced rotation rates of at least eight ipsilateral turns per min (Fig. 4B). At 2, 3 and 6 weeks post-grafting, animals that had received an intrastriatal graft of E14 rat VM, together with either saline (‘graft alone’), rhGDF5 (50 μg) or 5 × 10^5^ GDF5-CHO cells with CyA displayed significantly lower rotation rates than at the pre-lesion testing-point and than the rates displayed by rats that had received a sham graft, or a co-graft of E14 VM and 5 × 10^5^ GDF-CHO cells without CyA (*P* < 0.001; Fig. 4B). Immunocytochemical analysis at 7 weeks post-grafting showed that there were significantly more dopaminergic neurons in the grafts that had received rhGDF5 or had been co-grafted with GDF5-CHO cells with CyA than in control grafts or those that had been co-grafted with GDF-CHO cells without CyA, or mock-CHO cells with or without CyA (*P* < 0.01; Fig. 4C and D). Immunocytochemical analysis showed that the dopaminergic neurons in E14 VM transplants that had been co-grafted with GDF5-CHO cells and CyA were more morphologically mature than those in control grafts. Specifically, the neurons in the co-transplants displayed longer neurites (mean total neurite per cell 23.6 ± 7.1 μm) when compared with those in grafts that had been implanted alone (mean total neurite per cell 10.8 ± 3.4 μm) (see Fig. 4E).

## Discussion

This study investigated the effects of long-term delivery of the neurotrophic factor, GDF5, on the lesioned adult rat nigrostriatal dopaminergic pathway and on transplanted embryonic rat dopaminergic neurons. It used a CHO cell line that had been modified to overexpress GDF5, to introduce a continuous supply of this neurotrophic factor to the adult rat brain. Thus, it built upon previous studies showing neuroprotective [26, 27] and restorative [25] actions of GDF5 treatment on the lesioned adult rat nigrostriatal pathway, as well as survival-promoting effects of GDF5 on embryonic rat dopaminergic grafts [21, 28].

Initial characterization of the GDF5-overexpressing cell line demonstrated secretion of the mature dimeric form of GDF5 *in vitro* for at least 15 days. Treatment of E14 rat VM cultures with conditioned medium from GDF5-overexpressing CHO cell cultures induced a two-fold increase in dopaminergic neuronal survival. This treatment had no effect on the total number of cells or total number of neurons in these cultures, indicating that the effects of GDF5 were specific to dopaminergic neurons. Importantly, this experiment proved that the GDF5 protein that was secreted from the engineered CHO cells was biologically active, and had protective effects on cultured dopaminergic neurons that had been predicted based on previous studies using recombinant GDF5 protein [20].

The next part of the study examined the feasibility of intrastriatal implantation of the CHO cell line into the striata of adult rats. As the CHO cells are effectively xenografts, initially a strategy to control the host immune response to the implanted cells was conducted. Consideration of the host immune response to grafts is pertinent to all studies on embryonic tissue transplantation in PD patients. In clinical studies, patients are routinely administered oral cyclosporine for at least 6 months following intraputaminal grafting of human foetal VM tissue. However, a host immune response to such allografts has been described, even in immunosuppressed patients, comprising the presence of several immune cell markers within the graft site upon post-mortem analysis [34]. Although the grafted dopaminergic cells appeared to be healthy in these patients, the presence of such an immune response may present a threat to the long-term viability and functioning of the grafts. The host immune response in adult rats after intrastriatal transplantation of allogenic neural tissue has previously been described [32]. The present study used the scoring mechanism described by Duan *et al*. [32] to compare the host immune response with intrastriatal grafting of the CHO cell line, in rats that had been treated with oral cyclosporine and in control rats. These responses were also compared with that to E14 rat VM allografts, the standard type of graft used in rat models of PD. The immune response, measured in terms of the intensity of immunostaining for CD 4, CD 8, MHC I, MHC II, IgM and Ed 1, was significantly higher in the striata of rats that had received CHO cell grafts alone than in those that had received CHO cell grafts and oral cyclosporin. In general, the immune response was greatest after 5 and 10 days and tended to decrease by 21 and 42 days; this decrease was most pronounced in the immunosuppressed animals. The host response to E14 VM allografts (in rats that had not received cyclosporin) was generally the same magnitude as that to CHO cells with cyclosporin, although in some cases it was larger, especially at the later time-points. This study showed the value of immunosuppression for decreasing the likelihood of host-derived rejection of CHO cell transplants, and thus all animals were administered oral cyclosporin in the following experiments.

Having established a need for immunosuppression, subsequent *in vivo* experiments showed that secretion of the mature dimeric form of GDF5 by the GDF5-CHO cell line could be achieved for at least 1 week *in vivo* following transplantation to the striatum of cyclosporin-treated adult rats. In the context of the adult rat models of PD used in this study, GDF5 delivery for 1 week should be sufficient to achieve neuroprotective effects on the lesioned or degenerating nigrostriatal system.

Two sets of *in vivo* experiments were performed to evaluate the effects of the GDF5-overexpressing CHO cell line in adult rat models of PD. The first showed that implantation of GDF5-CHO cells could significantly decrease the neurodegeneration induced by a complete 6-OHDA lesion of the MFB. The 6-OHDA MFB lesion is an acute neurotoxic insult leading to the very rapid death of midbrain dopaminergic neurons. Lesion-induced motor asymmetry and loss of nigral dopaminergic neurons were both prevented by simultaneous implantation of the GDF5-overexpressing cell line into the ipsilateral striatum. These data demonstrate that sustained delivery of GDF5 *in vivo* can prevent the loss of dopaminergic neurons in the 6-OHDA MFB model of PD.

The second experiment used the ‘partial lesion’ model of PD, which involves 6-OHDA administration to the dopaminergic terminals in the adult rat striatum. This induces rapid degeneration of striatal dopaminergic fibres, followed by the protracted death of their nigral cell bodies, which begins after a delay of about a week and progresses over several weeks [35–39]. Thus, this subacute neurotoxic insult leads to the slower, more protracted death of dopaminergic neurons than that observed in the MFB lesion model, and is a better model of the progressive nature of the neurodegeneration that occurs in PD. Implantation of the GDF5-CHO cells at 1 week after the lesion allowed the protective effects of this intervention to be assessed at a time, when the degeneration was still ongoing. As shown above, these cells were capable of delivering GDF5 *in vivo* for at least a week after implantation. Implantation of the GDF5-overexpressing cells into either the striatum or midbrain, ipsilateral to the lesion, resulted in significant improvements in rotational behavioural performance, an indicator of striatal dopaminergic terminal integrity, and in survival of nigral dopaminergic cell bodies. This shows that in a situation where midbrain dopaminergic neurons are undergoing progressive cell death, sustained delivery of GDF5 *in vivo* to either the striatum or SN can protect these neurons from the ongoing degeneration. The more pronounced effect of CHO-GDF5 cells on nigral cell survival in the MFB lesion study compared with that in the striatal lesion study likely reflects the fact that the former is a neuroprotective treatment (applied at time of lesion), whereas the latter induces ameliorative effects on an already-degenerating nigrostriatal system (applied after lesion).

Recent studies have used cells, such as neural progenitor cells [40, 41] and bone marrow stromal cells [42], to overexpress the related neurotrophic factor, GDNF, in rat models of PD. Akerud *et al*. reported that transplanted GDNF-overexpressing neural stem cells survived well in the 6-OHDA-lesioned mouse striatum, expressed high levels of GDNF for at least 4 months, and had protective effects on the host nigral dopaminergic neurons [40]. Another study found that GDNF-expressing human neural progenitor cells could survive and release GDNF for at least 8 weeks after transplantation into 6-OHDA-lesioned rats and aged monkeys [41]. That study showed behavioural improvements in the parkinsonian rats, and a strong trend towards functional improvements in the aged monkeys. A more recent study reported neuroprotective effects of GDNF-expressing bone marrow stromal cells after intrastriatal transplantation in a lactacystin-treated mouse model of PD [42]. The present study has shown cell survival and functional effects which compare well with these previous studies. However, the use of cell lines, such as the CHO line, is not optimal because of the need for immunosuppressive treatment of the host. More appropriate cell types for sustained GDF5 delivery, such as embryonic and neural stem cells, are now under investigation in our laboratory. Nevertheless, the current study supports the proposed application of GDF5 in PD therapy and paves the way for the development of safer mechanisms for the sustained delivery of this neurotrophic factor to the parkinsonian brain.

The final study focused on evaluating the effects of intrastriatal co-implantation of GDF5-overexpressing CHO cells with embryonic rat VM cells, in rats with MFB lesions. A previous study involved the co-transplantation of primary foetal grafts and GDNF-producing neurospheres, prepared from embryonic rat striatum, into adult rats that had MFB 6-OHDA lesions [43]. That study found that the survival of the foetal grafts was enhanced by co-implantation of the GDNF-producing neurospheres, but that this was not reflected by any behavioural recovery in the animals. The authors attributed the lack of behavioural effects to either down-regulation of the transgene expression over time or death of the transplanted cells. In the present study, significant improvements in amphetamine-induced rotational behaviour was observed in all rats that had received a control graft, a GDF-treated graft or a CyA-treated graft co-implanted with GDF-CHO cells, whereas no changes were seen in the rats that had received co-implants without CyA. This shows that in the rats which were treated with CyA, the GDF5-overexpressing cells survived well and did not induce any detrimental effects on the grafted VM cells. However, there was no difference in the degree of reversal of amphetamine-induced rotational asymmetry between the groups that had received untreated grafts, GDF5-treated grafts and GDF5-CHO-co-implanted grafts. This probably reflects the fact that there were enough surviving dopaminergic neurons in each of these grafts at the 6-week time-point to ensure almost complete reversal of rotational asymmetry. Post-mortem immunohistochemistry showed that each of the rats in these three groups had more than 4000 surviving dopaminergic neurons. It has been shown that ∼2000 transplanted dopaminergic neurons can achieve a 50% reduction in amphetamine-induced rotations [44]. In animals that had been treated with cyclosporine, VM grafts that were co-implanted with GDF5-CHO cells were found to have significantly more dopaminergic neurons than control VM grafts or those that were co-implanted with mock-CHO cells. In fact, the dopaminergic neuronal survival in GDF5-CHO treated grafts was equivalent to that in VM grafts that had been treated with a high dose (50 μg) of recombinant human GDF5. In addition, upon post-mortem examination, the dopaminergic neurons within these treated grafts were more mature than those in control grafts, displaying longer neurites and more branching. An effect of GDF5 on dopaminergic neuronal morphological development has been previously shown [20–22] and may be critically important for the use of this factor in grafting strategies, where the successful integration of the transplanted dopaminergic neurons into the host striatal neuronal circuitry is necessary to confer functional effects. Transplantation of embryonic dopaminergic neurons or those derived from other sources, such as stem cells, remains a viable option for the treatment of PD [7, 45]. The success of this approach will ultimately be determined by the survival of the transplanted cells and their integration into the host brain parenchyma. The current data show that sustained delivery of GDF5 *in vivo* increases the survival of transplanted dopaminergic neurons and improves their morphological development, which may promote their functional integration into the host striatum.

Two of the most promising strategies for the future therapy for PD are the delivery of neurotrophic factors to protect degenerating dopaminergic neurons, and the use of embryonic dopaminergic neurons or those derived from other sources, such as stem cells, to replace the lost dopaminergic neurons. The current study has shown that overexpression of the neurotrophic factor, GDF5, has protective effects on the degenerating adult rat nigrostriatal system and survival-promoting effects on transplanted embryonic rat dopaminergic neurons in an *in vivo* rat model of PD. The use of xenogenic cell lines, such as CHO cells, is not feasible for clinical application because of immunogenic concerns and the risk of tumour formation. Thus, the development of safer methods of delivering neurotrophic factors in appropriate doses to the degenerating nigrostriatal dopaminergic system is paramount to the future successful clinical application of neuroprotective therapies for PD. In conclusion, this study has provided proof-of-principle that sustained delivery of GDF5 *in vivo* can confer neuroprotective effects in PD models, and it paves the way for the development of efficient, targetted and safe delivery methods.
